# Clinical Features and Treatment Outcomes of Primary Immune Thrombocytopenic Purpura in Hospitalized Children Under 2-Years Old

**Published:** 2016-03-15

**Authors:** H Farhangi, A Ghasemi, A Banihashem, Z Badiei, L Jarahi, G Eslami, T Langaee

**Affiliations:** 1 **Department of Pediatric Hematology and Oncology, Dr Sheikh Pediatric Hospital, Mashhad University of Medical Sciences (MUMS), Mashhad, Iran. **; 2 **Department of Community Medicine, Mashhad University of Medical Sciences, Mashhad, Iran. **; 3 **Department of Pharmacotherapy and Translational Research, Center for Pharmacogenomics, College of Pharmacy, University of Florida, USA.**; 4 **Department of Pharmacotherapy, College of pharmacy, Mazandaran University of medical sciences, Sari, Iran.**

**Keywords:** ITP, Intravenous Immune Globulin, Pediatric, Platelet, Vaccination

## Abstract

**Background:**

Immune thrombocytopenic purpura (ITP) is the most prevalent cause of thrombocytopenia in children. Despite the importance of ITP in children under 2-years old, only a few publications are available in the literature.ITP usually presents itself as isolated thrombocytopenia and mucocutaneous bleeding.

**Materials and Methods:**

This study was conducted on 187 under 2-year-old children diagnosed with ITP and treated at Dr. Sheikh Hospital from 2004 to 2011.In this retrospective study, clinical symptoms, laboratory findings, history of viral infections, vaccination history, and treatment efficacy in children under 2-years old with ITP were investigated.Patients were followed for one year after being discharged from the hospital.

**Results:**

The risk of the disease developing into chronic form was higher in older children (0.001). ITP in children under 3-months old was significantly associated with vaccination (p=0.007). There was no significant differences between male and female patients in regards to newly diagnosed ITP, persistent, and chronic disease status (p = 0.21). No significant difference in bleeding symptoms was observed between patients under 3-months old and 3 to 24-months old (p=0.18).

**Conclusion:**

Infantile ITP respond favorably to treatment. The risk of the disease developing into chronic form is higher in 3-to-24-month-old children compared to under-three-month olds.

## Introduction

Immune thrombocytopenic purpura (ITP) is an autoimmune bleeding disorder in children, presenting itself with petechiae, easy bruising, and mucosal bleeding (1). ITP is characterized by isolated thrombocytopenia (platelet count of <100,000/ul with normal white blood cell count and hemoglobin(1-3).While the acute form of ITP often follows an infection and usually resolves spontaneously within 12 months, chronic ITP persists longer than 12 months without a specific cause. The cause of ITP is still unknown in most cases, but it can be triggered by vaccines or exposure to viral antigens via respiratory or gastrointestinal infections (1, 4, 5). Although the disease is more common in children aged 2 to5years old, it occurs in other age groups as well (6-8). In children with ITP, the risk of serious bleeding and intracranial hemorrhage (ICH) is about 3 and 0.5 percent, respectively. It remains unproven that pharmacologic therapy reduces the risk of ICH and other life-threatening complications of ITP. Approximately 20 percent of affected children will develop chronic ITP, defined as ongoing thrombocytopenia, lasting more than12 months from presentation (9-11). As the disease resolves spontaneously in many cases, treatment is rather of a more prophylactic type, with medical intervention necessary only in cases where dangerously low platelet counts increases the risk of life-threatening spontaneous bleeding. Standardized treating options include corticosteroids, immunoglobulins and anti-D antibodies in various regimens (8). Infantile ITP is reported to be characterized by different clinical features including: higher prevalence in male patients, lower rate of chronicity, less frequently preceded by infection, more severe clinical course and less favorable response to treatment(5, 12).Despite the importance of ITP in children under 2-years old, a few publications are available in the literatures.The current study sought to undertake a retrospective study to investigate the clinical symptoms, laboratory findings, history of viral infections, vaccination history, and treatmentefficacy in children under 2-years old with ITP.

## Materials and Methods

This retrospective study was conducted on 187 under2-year-old children diagnosed with ITP in Dr.Sheikh Pediatric Hospital (A 150 bed referral-teaching hospital in the East of Iran) for a period of 8 years between 2004 and 2011.Informed consent was obtained as required by localinstitutional review boards. Data including age, sex , history of vaccination over the past six weeks (according to vaccination program), patients' clinical symptoms, laboratory test results, treatments, patients’ response to treatment, history of respiratory infections, and diarrhea in the last two weeks were extracted from patients' records.Bone marrow aspiration(BMA) was performed in all patients. Patients were followed for one year after being discharged from hospital (data was collected on bleeding episodes during the6-month-period such as, intracranial hemorrhage (ICH) or whetherthe patient received any platelets or not). Patients were also divided in 3 main categories based on different treatment strategies: 1) parenteral corticosteroids injections (methylprednisolone 20-30 mg/kg or dexamethasone 10mg/m2), 2) oral corticosteroids (prednisolone 2mg/kg daily), 3) corticosteroids + IV Intravenous Immunoglobulin (IVIG) (1-2 g/kg IV).

**ITP Diagnosis**

The diagnosis of ITP was determined by the physicians based on history and physical examination; complete blood count presentedisolated thrombocytopenia (platelet count <100,000), normal hemoglobin, white blood cell count, peripheral blood smear, absence of underlying conditions such asfamilial thrombocytopenia, HIV, systemic lupus erythematosus, or malignancy. Bone marrow aspirate and serological studies were used to rule out infectious and rheumatic diseases.Coagulation studies were also performed as clinically indicated.Eventually, the diagnosis was confirmed bybone marrow aspiration (BMA). Among 218 children younger than 2years, 31 were excluded because of the other causes of thrombocytopenia. The remaining 187 patients formed the basis of the present study. Mothers’ complete blood count (CBC) and infants’ CBC at delivery were considered for ruling out secondary to maternal thrombocytopenia.

**ITP Classification**

Chronic ITP was defined as cases with platelet count of less than 100,000/µl for more than 12 months despite treatment. Persistent ITP was defined in patients with duration of thrombocytopenia between 3 to 12 months from diagnosis. Newly diagnosed ITP was defined as thrombocytopenia within 3 months from diagnosis. Active mucosal bleeding was defined as bleeding from the nose and gums, gross hematuria, gastrointestinal bleeding, nd brain hemorrhage. Patients were divided into less than 3-months and 3 to 24-months old age groups andthe collected data were compared between them.Response to therapy was defined as platelet count of ≥ 30 × 10^9^/L and at least 2-fold increase in the baseline platelet count and absence of bleeding.Complete response (CR) to treatment was reported when platelet count was ≥ 100 × 10^9^/L and absence of bleeding (1, 5). 

**Statistical Analysis**

The data were analyzed by SPSS (version 11.5) and the association between qualitative variables was performed by chi-square and Fisher’s exact test. Mean differences between quantitative variables in the case of normal distribution were determined by T -test and ANOVA. Correlation test was used to investigate the association between quantitative variables with each other. P-value of less than 0.05 was considered as significant. 

## Results

**Demographics data**

A total of 187 patients with ITP were reviewed in this study. There were 68 (36.36%) female and 119 (63.64 %) male patients with mean age of 190.82 ± 208.24 days and median age of 75 days (range from 17-720 days). Of the total number of patients reviewed, 112 (60%) were younger than 3-months, and 75 (40%) were older (TableIII). The highest prevalence rate of the disease was in October (11.2%), and the lowest rate occurred in January (2.7%). Most newly developed infantile ITP cases were observed in summer. 

**Clinical Features and Laboratory Values**

Petechiae and purpura were the most commonly observed symptoms present in 144 (77%) patients.Other symptoms such as epistaxis, gastrointestinal bleeding, gum bleeding, cerebral hemorrhage, and other type of bleeding were present in 17 (9.09 %), 13 (6.95 %), 6(3.2 %), 3 (1.6 %), and 4(2.13 %) of patients, respectively. Bleeding symptoms were observed in 43 (23%) patients. The history of vaccination in the past 6 weeks was positive in 90 (48.12%) patients. The history of upper respiratory tract infections in the past 2-weeks, and the history of diarrhea were observedin 57 (30.48%) and 14 (7.48%) patients, respectively (Table I). All the laboratory data for patients including platelet count before and after treatment, hemoglobin (Hgb) level, and white blood cell (WBC) count are shown in Table II.

**Treatment outcomes**

**Figure1:**
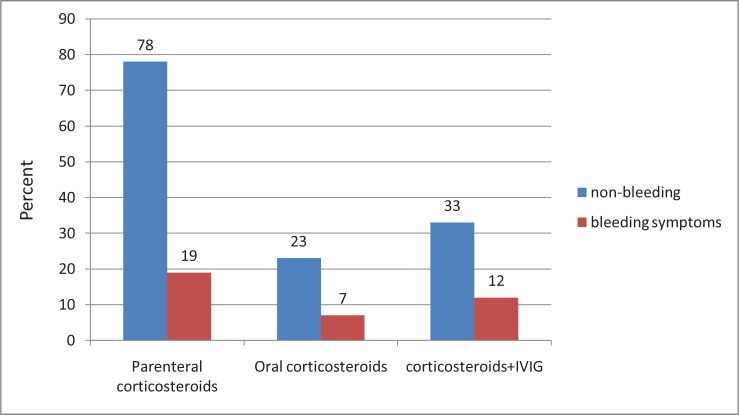
Different treatment methods used in children with ITP considering bleeding or non bleeding symptoms

Different methods of treatment were divided into three groups as shown in Figure 1. Parenteral steroids were the most commonly used therapeutic drugs.There was no difference in mean platelet count among various treatment groups at admission. All treatment modalities resulted in statistically significant platelet increase (P = 0.0001). The mean age of the oral steroids treatment group was significantly higher than other treatment methods (p = 0.023), 270 ± 301 days vs. 185 ± 183 and 179 ± 141 days for the parenteral steroid and steroid + IVIG, respectively. There was no significant difference between male and female patients in regard to complete response, persistent, and chronicdisease status (p=0.92). Admission platelet count did not show any significant relation with the length of hospital stay (p = 0.27).

**Bleeding symptoms and contributing factors**

There was no difference between bleeding and non-bleeding groups based on gender (P = 0.66). The history of gastroenteritis had significant impact on bleeding symptoms (0.004).The method of treatment was not associated with bleeding symptoms (p = 0.60).

**Acute and Chronic ITP**

The presence of chronic disease did not differ significantly in male and female patients (p = 0.92).The history of respiratory infections (p = 052), diarrhea (p = 0.38), and vaccinations (p = 0.22) was not associated with chronic disease. The frequency of bleeding symptoms did not differ in acute and chronic forms of the disease (P= 0.27). The mean age of patients with acute and chronic ITP was 166.2 ± 188 and 533.8 ± 186.2days, respectively. The mean age of patients in two groups was significantly different (P=0.0001), and chronic ITP was more common in older patients (Table III)*.*

**History of vaccination in ITP**

History of vaccination in past 6 weeks did not have any effect on platelet count (p = 0.28), hemoglobin level (p = 0.51), and duration of hospitalization (p = 0.51). ITP in children under 3-months was significantly associated with vaccination (p=0.007). The mean age in positive history of vaccination was127.5 ± 137.5 days, and in patients without any history of vaccination was 249 ± 243 days (P= 0.0001). 

**ITP in different age groups**

The laboratory tests, mean length of hospital stay, patients’ history of vaccination, and infections for all patients are shown in Table III.No significant difference was observed in the prevalence rate of bleeding symptoms (p = 0.18) in under-3-month-olds (22 patients, 19.6%) and 3-to-24-month-olds (21 patients, 28%). The most common symptom upon admission in both age groups was cutaneous manifestations. The most frequently observed symptom of the disease in both age groups was reported to be skin manifestations, with the prevalence rate of 79.5% (89 patients) and 72% (54 patients) in under-3-month-olds and 3-to-24-month-olds, respectively. Cerebral hemorrhage only occurred in 3 patients under 3-months old.

**Table I:** *Demographic data and clinical symptoms upon admission*

Varibles	Number ( %) or mean± SD
Age (days )	190.82 ± 208.24
Gender	
Female Male	68 (36.36%) 119 (63.64%)
Vaccination History (past 6-weeks)	
Polio + hepatitis Polio + hepatitis + DTP Polio + DTP Polio + DTP + MMR	17 (9.1%) 58 (31%) 4 (2.1%) 11 (5.9%)
History of viral infection (past 2-weeks)	
URI Gastroenteritis	57 (30.48%) 14 (7.48%)
Duration of hospitalization (days)	4.39±2.29
Outcome	
Complete response Persistent Chronic disease	167 (89.3%) 8 (4.27%) 12 (6.41%)
Symptoms	
Petechiae and purpura Cutaneous symptoms + gastrointestinal bleeding Cutaneous bleeding + nasal bleeding Cutaneous bleeding + gum bleeding Cutaneous bleeding + cerebral hemorrhage Others	144 (77%) 13 (6.95%) 17 (9.09%) 6 (3.2%) 3 (1.6%) 4 (2.13%)
Treatments Methods	
Parenteral corticosteroids Oral corticosteroids Corticosteroids + IVIG Others	97 (51.87%) 30 (16.04%) 45 (24.06%) 15 (8.02%)

** DTP:** Diphtheria, Pertussis, Tetanus; **MMR:** Measles, Mumps, Rubella; **IVIG: **Intravenous Immunoglobulin

**Table II:** *Laboratory Values of pediatric patients with ITP*

**Variable**	**Mean**	**Median**	**Range**	**Mode**
Platelet count( /µl) Baseline After treatment	11521 133647	10000 104000	1000-62000 12000-753000	3000 25000
Hemoglobin (g/dl)	10	10	6-20.1	10.7
White blood cell( /µl)	9297	8700	3900-18600	7600

**Table III:** *Comparing Baseline data and outcomes in different age group*

**Variable**	<3months	>3months	P-value
Number (%)	112 (60%)	75 (40%)	
Platelet count ( *10 ^3^ ) At admission^¥^ At discharge ¥	11.178 ± 8.369 146.86 ± 120	12.033 ±9.966 113.9 ± 99	0.50 0.05*
Hemoglobin (g/dl) At admission ¥	9.5 ± 1.7	10.6 ± 1.2	0.001*
Length of hospitalization (days) (Mean± SD)¥	4.5 ± 2.4	4.1 ± 2.1	0.22
Upper respiratory infection in past 4-weeks£	29 (25.9%)	28 (37.5%)	0.096
Diarrhea^α^ (past 2-weeks)	6 (3.2%)	8 (4.3%)	0.17
Vaccination£ ( past 6-weeks)	63 (33.7%)	27 (14.4%)	0.007*
Chronic disease^α^	0 (0%)	12 (16%)	0.0001*

* P value < 0.05

¥ : independent sample T test

£ : chi^2 ^test Α: fisher exact test

## Discussion

Characteristics and features of childhood ITP have been extensively studied, but not 

in infants or children younger than 2 years old (1, 7, 13). In an effort to better characterize this patient population, this retrospective study was conducted. Ballin et al., described 57 patientsyounger thanyears of with acute ITP (13).Hord et al., and Sandoval et al., also reported ITP in 12 and 79 pediatric patients, respectively (1, 14). In patients between ages of 2 and 5 years, the disease was more frequent in males with less history of infectious disease, poorer response to treatment, lower chance of chronicity, and more severe clinical symptoms (1, 5, 14). ITP was also more prevalent in male patients. So far, there is no explanation for the higher frequency of ITP in male subjects (5). Severity of bleeding symptoms was different between age groups (4, 6, 12). The incidence of bleeding symptoms in patients under 3-months and older than 3-months were 19.6% and 28%, respectively, compared to 17-27% risk of bleeding previously reported in children with ITP(1). Most of the patients (89.3%) responded completely to the treatment, which is in concordance with the results reported in other studies (2, 6, 7, 15). In contrast, Ballin et al., in a retrospective study reported 30% risk of chronic ITP in 57 male infants with lack of responsiveness to treatment. Hord et al., described ITP in 12 infants of 1 to 12-months old, where one infant suffered from chronic ITP and no serious bleeding complications were observed ((13, 14). The study by Sandoval et al., showed that chronic ITP was more common in older age with risk of about 9% (1). It’s been shown that vaccination (MMR, hepatitis, varicella) is related to ITP (4, 6-8, 13-15). Miller et al., for instance studied the risk of ITP recurrence after MMR vaccination. The risk in patients who had received the vaccine in the past 2 to 6 weeks was reported to be 3.27 times more than the control group (16). Similarly, Clara et al. also reported a 71% accompaniment of past-two-months vaccination with the recurrence of the disease in nineteen under-one-year-old children with ITP (6). In the current study, 47% of children with ITP had history of vaccination in the past six weeks; of this 47%, 11 patients had already a history of MMR vaccination. Although the present study showed an association between vaccination and ITP emergence, Sean et al. showed that vaccination does not increase the risk of ITP, the only exception being MMR (15). Despite reports about the risk of ITP recurrence after MMR re-vaccination, ACIP (the AmericanCommittee of Immunization Practices) does not strongly contraindicate a second dose of MMR in children with a history of ITP after the first dose (1). In the present study, immunization was administered according to the national vaccination program of Iran, except in cases where the patient had received IVIG treatment. In these cases, the administration of live attenuated vaccines was postponed for 4 to 6 months.

Active mucosal bleeding was reported in 8% of the patients in a study conducted by Sandoval (1). In the current study 23% of the patients showed active bleeding symptoms. This difference may be due to the greater number of inpatients in this study (Sandoval et al.’s selected patient population included both inpatients and outpatients). The present study did not show any significant difference between the risk of active mucosal bleeding in the under-three-months patients and the other age group (3 to 24 months). The risk of intense bleeding and mucosal bleeding reported in other studies are 17% and 26-27% respectively (1, 4, 5, 12). The most common treatment method in the current study was parenteral steroids and the mean age of patients was highest in the oral steroids group. IVIG and steroid were prescribed in lower age group. The more prevalent use of parental steroids in infants may be due to difficulty in taking tablets and concern about hemorrhage. The lower use of IVIG in older children was related to the cost of IVIG and the need for higher dose of drug based on their body weight. Kalyoncu et al., showed the superiority of methylprednisolone to IVIG regarding the efficacy, side effects, availability, and cost (12). Since IVIG increased the platelet count more rapidly than corticosteroids in the first 48 hours of treatment (17), it could be a good choice in patients with severe bleeding upon admission or in patients with high risk of cerebral hemorrhage. The use of IVIG in patient was reasonable considering the cerebral hemorrhage in infants less than 3-months old. In general, personal preferences (patients’ parents) and physicians’ choice played a major role in choosing the treatment for ITP. An increased rate of hospitalization and use of IVIG in infants were observed because parents and physicians were aware of the risk of serious bleeding in this age group (1, 6, 17). The incidence of acute ITP was reported to be more common in spring and winter (4, 6, 15). In contrast, the study by Kalyoncu et al., in Turkey reported the most incidences of ITP in summer (9). In this study, the most prevalence of ITP was in summer; also the prevalence was significantly high upon seasonal changes. Moreover the peak of gastroenteritis during the months of July and August were associated with more cases of ITP in children. Positive history of infection in infantile ITP was reported to be 57.1% by Clara Lo et al. (6); This rate was reported to be 52% by Thomas Kühne (5). In the current study, the total prevalence rate of respiratory and gastrointestinal infections was 37.7%. This rate was not significantly different in the two age groups (under-three-month-olds and 3-to-24-month-olds). The lower prevalence rate of prior infection in this study may be due to the selected patient population (bigger population of under-three-month-olds and in patients).

## Conclusion

Infantile ITP respond favorably to treatment. The risk of the disease developing into chronic form is higher in 3-to-24-month-old children compared to under-three-month olds.
